# Prevalence and risk factors of intra-dialytic hypotension: a 5 year retrospective report from a single Nigerian Centre

**DOI:** 10.11604/pamj.2017.28.62.13743

**Published:** 2017-09-21

**Authors:** Ogochukwu Chinedum Okoye, Henry Enyinmisan Slater, Nilum Rajora

**Affiliations:** 1 Nephrology Division, Department of Medicine, Delta State University Teaching Hospital, Nigeria; 2 Nephrology Division, UT Southwestern Medical Centre, Dallas Texas, USA

**Keywords:** Intra-dialytic hypotension, haemodialysis, risk factors

## Abstract

**Introduction:**

Intra-dialytic hypotension (IDH) is a common complication of haemodialysis that impacts negatively on the patient's quality of life and can induce serious cardiovascular events.

**Methods:**

Records of all adults who had haemodialysis treatments from Jan 2012-Jan 2016 were reviewed. Socio-demographic data, health status of patient, aetiology of renal disease, clinical and biochemical parameters such as systolic and diastolic blood pressures (SBP and DBP), packed cell volume, were collated using Microsoft Excel.

**Results:**

The overall prevalence of intra-dialytic hypotension was 8.6%. Of all haemodialysis patients, 45.7% experienced a drop in SBP > 20mmHg, 28.5% required nurses' intervention and 8.6% had symptoms. Diagnosis of obstructive nephropathy (OR: 3.1, CI:1.43-6.60, p = < 0.004) and sepsis (OR: 3.57, CI: 1.31- 9.75, P = 0.013) increased the odds of experiencing IDH. Only 5% of patients with predialysis SBP < 100mmHg developed IDH (OR: 0.12, CI: 0.02-0.93, P = 0.04).

**Conclusion:**

IDH was common among the patients studied. It was more prevalent among patients with obstructive nephropathy and sepsis; however other traditional risk factors of IDH such as older age and anaemia, were not found to be significantly associated with IDH. Surprisingly, prevalence of IDH was significantly less among patients with pre-dialysis hypotension compared to those without.

## Introduction

Haemodialysis is one of the cornerstones of management of chronic kidney disease in Nigeria [1]. Although haemodialysis is a relatively safe procedure, a number of complications may arise which includes intradialytic hypotension (IDH). There is no generally accepted definition for IDH [2]. The Kidney Disease Outcome Initiative (K/DOQI) defined IDH as a decrease in systolic blood pressure by > 20mmHg or a decrease in mean arterial pressure by 10mmHg, associated with symptoms that include abdominal discomfort, yawning, sighing, nausea, vomiting, muscle cramps, restlessness, dizziness, fainting and anxiety [3]. The European Best Practice Guidelines (EBPG) [4] definition is slightly modified to include presence of symptoms and need for nurses intervention. The prevalence of IDH varies from 20-50%, due to inconsistent definitions used across studies. The incidence of IDH is 25% in the US [5]. Amira et al [6] in South-west Nigeria, reported that IDH (defined using EBPG) complicated 8.5% of 1010 haemodialysis treatments. IDH was found to be significantly commoner with initial treatment (25.9%), in older patients, patients with obstructive uropathy (probably due to their older age) and those with low to normal blood pressures. Kuipers et al [7], in a prospective study and applying the EBPG guideline, reported that there was a significant SBP or MAP drop in 77% of the 3818 haemodialysis treatments studied. In same study, 21.4% had intradialytic events and only 6.7% required nursing intervention, resulting in an overall IDH prevalence of 8.5%; prevalence of nurses intervention being the main determinant of overall prevalence. Epidemiological study on IDH and its risk factors are generally scarce in the local literature.

Some of the risk factors of IDH in Chronic kidney patients include: diabetes mellitus, cardiovascular disease (CVD), poor nutritional status and hypoalbuminemia, autonomic dysfunction, severe anemia, age above 65 years and systolic blood pressure < 100mmHg [8] IDH is common and has been attributed variably to body volume depletion, shifting of fluid from extracellular to intracellular space [9], left ventricular hypertrophy and cardiac remodeling particularly in the CKD patients [10]. Patients with chronic kidney disease have defective reactivity of the resistance vessels and capacitance vessels during haemodialysis however; the exact mechanism for this is unknown [11]. In contrast, data from studies involving isolated ultrafiltration and haemofiltration have shown that vascular responses remained intact [12]. IDH impacts negatively on patient's quality of life and can induce cardiovascular events including cardiac arrhythmia, coronary or cerebral ischemic disease [13, 14]. Long term effects of IDH includes volume overload due to suboptimal ultrafiltration, use of boluses for resuscitation [3] and inefficient clearance due to adjustments in dialysis prescription to prevent IDH. The result is that some of these patients are forced to seek alternative and sometimes, harmful treatment for their symptoms, since they wrongly believe that haemodialysis either worsens or does not change their clinical state [15]. Some measures recommended to reduce the risk of IDH include: counseling patients to minimise interdialytic weight gain, discontinuing antihypertensives medications prior to dialysis, avoiding the use of long acting vasodilators, avoiding eating before and during procedure and echocardiographic evaluation of ESRD patients [16]. Furthermore treatment related interventions such as: avoiding excessive ultrafiltration, sodium profiling, isolated ultrafiltration and use of some medicines; have been recommended [16]. The aim of this study was to determine the prevalence and risk factors of intradialytic hypotension amongst haemodialysis patients in the study centre.

## Methods

A retrospective cross-sectional study carried out in a tertiary hospital in Southern Nigeria. Records of all patients who had haemodialysis treatments from Jan 2012-Jan 2016 were obtained from individual dialysis case folders. All adult haemodialysis patients with diagnosis of acute or chronic kidney disease, were included. Patients with incomplete or incorrect data were excluded. Socio-demographic data, health status of patients, aetiology of renal disease, some clinical and biochemical parameters such as systolic and diastolic blood pressures pre and post haemodialysis, packed cell volume (PCV), were collated using Microsoft Excel. A patient was considered as having IDH if systolic blood pressure drops by > 20mmHg anytime during a haemodialysis procedure, associated with clinical features and nurses intervention per EBPG guidelines [4]. Nurses intervention was regarded as use of intravenous fluids, blood products, need to adjust dialysis prescription or need for medications eg. vasopressors. Data were analyzed using statistical package for social sciences (SPSS) version 22.0 software (SPSS Inc. Chicago, Illinois, USA). The main analysis was the determination of the crude prevalence of IDH for the sample. A 2x2 table and Chisquare analysis was used to test for any statistically significant difference between IDH (outcome variable) and risk factors. The unadjusted Odds ratio was calculated for risk factors such as age > 65years, sex, aetiology of kidney disease, pre dialysis systolic blood pressure < 100mmHg and anaemia using univariate logistic regression analysis. A P value of < 0.05 was regarded as significant. For the tests of significance, IDH was defined using evidence of BP drop and nurses intervention; this was because both parameters were more consistently recorded in charts compared to patients symptoms.

## Results

Four hundred and four complete files were included in the data analyses. Majority (55.7%) were males with a sex ratio of 1.25:1. The mean age was 48 ± 17years and the mean pre-dialysis systolic blood pressure and diastolic blood pressure were 145 ± 32mmHg and 82 ± 20mmHg respectively. CKD was the diagnosis in 74.8% of patients, while 25.2% had acute kidney injury. The common aetiologies of CKD included: chronic glomerulonephritis (28.1%), hypertension (27.5%) Diabetes (19.2%), Obstructive nephropathy (10.9%), HIV associated Nephropathy (6.0%) amongst others; while the major causes of AKI were sepsis (44.1%), obstructive nephropathy (14.7%), hypovolaemia (12.7%) and ecclampsia (10.8%) (Table 1). The prevalence of IDH according to EBPG was 8.6% i.e of all patients studied, 8.6% experienced the combination of a drop in SBP > 20mmHg, characteristic symptoms and need for nursing intervention. However considering the criteria individually, 45.7% of all patients studied experienced a drop in SBP > 20mmHg, 8.6% had symptoms and 28.5% required nurses intervention. There was no statistically significant difference between males and females (20.6% vs. 18.9%, p = 0.716). IDH was commoner amongst patients with AKI compared to those with CKD (27.5 vs. 24.8% p = 0.128). A significantly higher proportion of CKD patients with Obstructive nephropathy developed IDH compared to those without (OR: 3.1, CI: 1.43-6.60, p = 0.004); 39.4% of those with obstructive nephropathy compared to 17.5% of those without obstruction, developed IDH. Only 3.4% of CKD patients with predialysis SBP < 100mmHg developed IDH compared to 27.8%% of those who had higher SBP (OR: 0.12, CI: 0.02-0.93, P = 0.04). A higher proportion of patients with HIVAN developed IDH compared to those without HIVAN (27.7% vs. 19.4%) and more patients with Diabetes nephropathy had IDH compared to those without (22.4% vs. 19.3%); however these did not reach statistical significance (Table 2). Among patients with AKI, sepsis increased the odds of experiencing IDH (OR: 3.57, CI: 1.31-9.75, P = 0.013).

**Table 1:** Aetiology of kidney disease among 404 patients studied

Aetiology	n (%)
**Chronic kidney disease**	
Chronic glomerulonephritis	85 (28.1)
Hypertension	83 (27.5)
Diabetes nephropathy	58 (19.2)
Obstructive nephropathy	33 (10.9)
HIVAN	18 (6.0)
Lupus nephritis	6 (2.0)
Sickle cell nephropathy	4 (1.3)
ADPKD	3 (1.0)
NSAID induced	3 (1.0)
*Others	9 (3.0)
TOTAL	302 (100.0)
**Acute kidney injury**	
Sepsis	45 (44.1)
Obstructive nephropathy	15 (14.7)
Hypovolaemia	13 (12.7)
Ecclampsia	11 (10.8)
Malignant Hypertension	4 (3.9)
Toxic nephropathy	4(3.9)
AGN	4(3.9)
Pyelonephritis	3(2.9)
Others (Acute Rejection, hepatorenal syndrome,	3(2.9)
TOTAL	102(100.0)

HIVAN = HIV associated nephropathy, ADPKD = Adult polycystic kidney disease, NSAID = Non-steroidal anti-inflammatory drugs, *= chronic pyelonephritis, renal cell carcinoma, multiple myeloma, lymphoma, and toxic nephropathy

**Table 2:** Risk factors of intra-dialytic hypotension (IDH) amongst CKD patients

Variable	IDH n (%)	NO IDH n (%)	OR (CI)	P value
Obstructive nephropathy	13 (39.4)	20 (60.6)	3.10 (1.43, 6.60)	0.004
Pre-dialysis Hypotension	1 (3.4)	29 (96.6)	0.12 (0.02, 0.93)	0.040
Male	37 (20.6)	143 (79.4)	1.11 (0.62, 1.99)	0.716
Female	27 (18.9)	99 (81.1)		
Age >65years	15 (24.6)	49 (75.4)	1.4 (0.73, 2.77)	0.302
AKI	22 (27.5)	80 (72.5)	1.11 (0.64, 1.92)	0.712
CKD	60 (24.8)	242 (75.2)		
HIVAN	5 (17.9)	23 (82.1)	0.83 (0.31, 2.38)	0.779
Anaemia (PCV <20%)	12 (18.5)	53 (81.5)	0.89 (0.44, 1.81)	0.759
Diabetes nephropathy	13 (22.4)	45 (77.6)	1.19 (0.59, 2.40)	0.609

AKI=acute kidney injury, CKD=chronic kidney disease, HIVAN=HIV associated nephropathy, PCV=packed cell volume

## Discussion

Intra-dialytic hypotension is a common complication of haemodialysis. CKD patients who had obstructive nephropathy and sepsis induced AKI were found to be at increased risk for IDH, however, traditional risk factors such as age > 65years, diabetes, SBP < 100mmHg and anaemia were not significantly associated with IDH. Surprisingly IDH was significantly less common amongst patients with predialysis systolic hypotension. The prevalence of IDH in this study, mirrors the findings of Amira et al [5] and Kuipers et al [6]. In this study presence of symptoms was the main determinant of the overall prevalence of IDH, rather than nurses intervention as was found by Kuipers et al. However, it is noteworthy that just as was reported by Kuipers and colleagues, a very high proportion of haemodialysis patients experienced significant drop in SBP during the procedure; in this study, 45.7% compared to 77% in the study by Kuipers. In the same study, 6.7% required nurse's intervention, compared to 28.5% in our study. Surprisingly, a higher proportion of patients received intervention (28.5%) compared to the proportion who experienced symptoms (8.6%); this tends to suggest that some interventions were based on blood pressure drop alone. Conversely, it is also probable that some patients received interventions for symptoms that were not recorded in the charts. Requiring clinical symptom and/or nurse's intervention as additional criteria to make a diagnosis of IDH tend to suggest that BP decline alone in these patients do not pose significant risk. Considering that some of these symptoms are subjective and patient's tolerance for certain symptoms vary, one can infer that some patients may actually not relay their symptoms to nurses, while others may exaggerate them. These afore-mentioned factors threaten the use of clinical symptoms as a compulsory criterion for diagnosing IDH. A major contributor to IDH is excessive ultrafiltration [2, 4] and in patients who already have other traditional risk factor(s) for IDH, the effect can be severe. Assessment of dry weight for individual patients is key to avoiding excessive UF. In this study, effect of inter dialysis weight gain was not investigated because of missing/incomplete record of patients' weight; a further contributory factor is that many haemodialysis patient are often too ill to stand on the available weighing scales. In the absence of an objective weight, the caregiver determines UF goal subjectively.

More reliable methods of assessing dry weight include bioimpedance spectroscopy, online continuous blood volume monitoring using haematocrit and protein measurements; measurement of some biomarkers like atrial natriuretic peptide, cyclic guanidine monophosphate (cGMP) amongst others [17]. These methods are not without limitations, however bioimpedance spectroscopy and continuous blood volume are useful easily practicable techniques [17], where the resources are available; unfortunately many centres in Nigeria do not practice these. IDH was commoner amongst patients with obstructive nephropathy; Amira et al [5] made a similar observation in the study. A plausible explanation is older age of the patients and presence of other comorbidities such, infections and malignancy. Not surprisingly, we observed IDH is commoner among patients with sepsis. These patients are usually quite ill in the setting of severe generalized inflammation, sometimes with multiple organ dysfunction including cardiovascular dysfunction resulting in haemodynamic instability. Some of our findings contrasts with what is known, for instance, the very low prevalence of IDH among patients with pre-dialysis hypotension seems to suggest that patients who have hypotension before treatment receive more aggressive, pre-emptive care to prevent IDH. In contrast, those who do not have pre-dialysis hypotension or other obvious risk for IDH, are managed less proactively. Although the risk for IDH is high in patients with pre dialysis hypotension, it is also known to occur in both normotensive and hypertensive patients [2, 4]. The other traditional risk factors of IDH were not significantly associated with IDH in the present study, this may be a reflection of possible overestimation of weight gain (due to the absence of reliable methods for assessing dry weight) with resultant excess ultrafiltration in a number of patients studied. This study was retrospective cross-sectional and so was limited by missing and incomplete information from source data. Furthermore this study design does not establish causation. Data for dry weight assessment (pre- and post dialysis weights) were mostly incomplete and therefore excluded from analysis; this may have provided useful information. Similarly, inconsistencies in recording patient's symptoms may have contributed to the low rates observed despite the higher proportions that experienced blood pressure drop and received intervention. Finally, prospective studies are preferable in this area of research, however a significant constraint in Nigeria is that many ESRD patients do not undergo dialysis beyond 1-3 month due to high costs of treatment and out-of pocket payment.

## Conclusion

IDH was common among the patients studied. It was more prevalent among patients with obstructive nephropathy and sepsis; however other traditional risk factors of IDH such as older age and anaemia, were not found to be significantly associated with IDH. Surprisingly, prevalence of IDH was significantly less among patients with pre-dialysis hypotension compared to those without. Thorough pre-assessment and monitoring of all patients during haemodialysis, remains crucial for effective prevention and treatment of IDH to ensure optimal dialysis and reduce associated morbidity and mortality. A simple checklist containing all possible risk factors of IDH as well as other plausible factors that could contribute to IDH should be developed and used to assess all patients before haemodialysis. Documentation of patient's complaints in dialysis treatment charts should be re-emphasized. Dialysis centres, especially in developing countries should be equipped with more reliable assessment tools for determining dry weight of dialysis patients, in the absence of this, dialysis beds or chairs with weighing scales incorporated, should be available.

### What is known about this topic

- IDH is a common complication of haemodialysis;
- Some of its risk factors include: diabetes mellitus, cardiovascular disease (CVD), poor nutritional status and hypoalbuminemia, autonomic dysfunction, severe anemia, age above 65 years and systolic blood pressure < 100mmHg.

### What this study adds

- One of very few studies that investigate IDH using recommended definition;
- Obstructive nephropathy and sepsis induced AKI are risk factors of IDH;
- The absence of reliable tools for assessing dry weight may significantly contribute to the high rate of IDH in developing countries and limit deductions from research in this field.

## Competing interests

The authors declare no competing interests.
